# Supplementary figures and images for: The Prevalence of Encephalitozoon cuniculi in Domestic Rabbits (Oryctolagus cuniculus) in the North-Western Region of Romania Using Serological Diagnosis: A Preliminary Study
Source: Microorganisms. 2024 Jul 16;12(7):1440. doi: 10.3390/microorganisms12071440 (PMC11279170; doi:10.3390/microorganisms12071440)

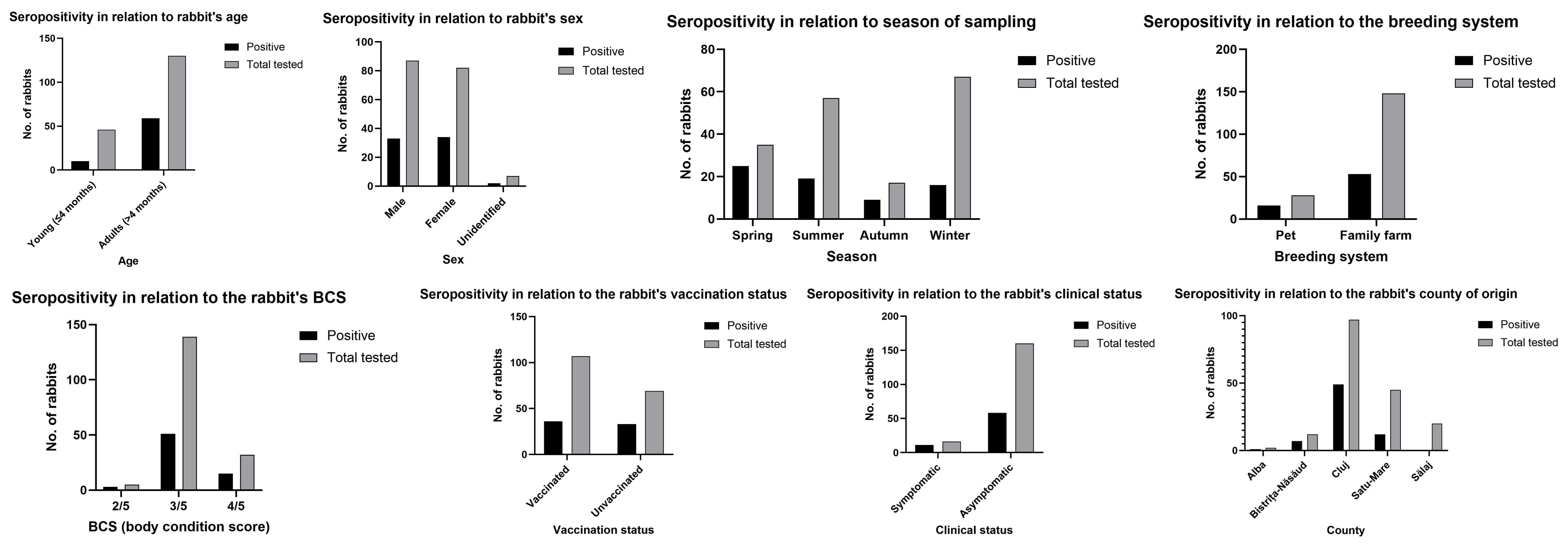

Supplement: Supplementary file 1 [file microorganisms-12-01440-s001.zip › microorganisms-3062955-supplementary.jpg]
